# Evaluation of the In Vitro Antimicrobial Efficacy against *Staphylococcus aureus* and *epidermidis* of a Novel 3D-Printed Degradable Drug Delivery System Based on Polycaprolactone/Chitosan/Vancomycin—Preclinical Study

**DOI:** 10.3390/pharmaceutics15061763

**Published:** 2023-06-18

**Authors:** Iván López-González, Ana Belén Hernández-Heredia, María Isabel Rodríguez-López, David Auñón-Calles, Mohamed Boudifa, José Antonio Gabaldón, Luis Meseguer-Olmo

**Affiliations:** 1 Tissue Regeneration and Repair Group: Orthobiology, Biomaterials and Tissue Engineering, UCAM—Universidad Católica de Murcia, Campus de los Jerónimos 135, Guadalupe, 30107 Murcia, Spain; ilopez27@ucam.edu; 2 Molecular Recognition and Encapsulation Research Group (REM), Health Sciences Department, UCAM—Universidad Católica de Murcia, Campus de los Jerónimos 135, Guadalupe, 30107 Murcia, Spain; abhernandez@ucam.edu (A.B.H.-H.); mirodriguez@ucam.edu (M.I.R.-L.); daunon@ucam.edu (D.A.-C.); 3 CRITT—Matériaux Innovation, 9 Rue Claude Chrétien, Campus Sup Ardenne, 08000 Charleville-Mézières, France; m.boudifa@critt-mi.com

**Keywords:** 3D printing, hybrid scaffold, polycaprolactone, chitosan, vancomycin, mesenchymal stem cells, tissue engineering, drug delivery systems (DDSs), osteomyelitis

## Abstract

Acute and chronic bone infections, especially those caused by methicillin-resistant *Staphylococcus aureus* (MRSA), remains a major complication and therapeutic challenge. It is documented that local administration of vancomycin offers better results than the usual routes of administration (e.g., intravenous) when ischemic areas are present. In this work, we evaluate the antimicrobial efficacy against *S. aureus* and *S. epidermidis* of a novel hybrid 3D-printed scaffold based on polycaprolactone (PCL) and a chitosan (CS) hydrogel loaded with different vancomycin (Van) concentrations (1, 5, 10, 20%). Two cold plasma treatments were used to improve the adhesion of CS hydrogels to the PCL scaffolds by decreasing PCL hydrophobicity. Vancomycin release was measured by means of HPLC, and the biological response of *ah*-BM-MSCs growing in the presence of the scaffolds was evaluated in terms of cytotoxicity, proliferation, and osteogenic differentiation. The PCL/CS/Van scaffolds tested were found to be biocompatible, bioactive, and bactericide, as demonstrated by no cytotoxicity (LDH activity) or functional alteration (ALP activity, alizarin red staining) of the cultured cells and by bacterial inhibition. Our results suggest that the scaffolds developed would be excellent candidates for use in a wide range of biomedical fields such as drug delivery systems or tissue engineering applications.

## 1. Introduction

Bone infections, such as osteomyelitis, remain a major clinical challenge in the field of bone surgery due to their serious rate of mortality and morbidity [1]. The most common causative species of surgical site infections and medical device-associated infections are the opportunistic Gram-positive staphylococci (≃75% of cases), particularly *Staphylococcus aureus* and *Staphylococcus epidermidis* [2,3]. In terms of pathogenesis, osteomyelitis is complex and varied, with bacteria reaching the bone in two ways: (i) endogenously via blood or originating from another nearby or distant source of infection (hematogenous osteomyelitis); and (ii) exogenously through direct inoculation, contamination of an open trauma, or postsurgical procedures [4].

**Figure 1:**
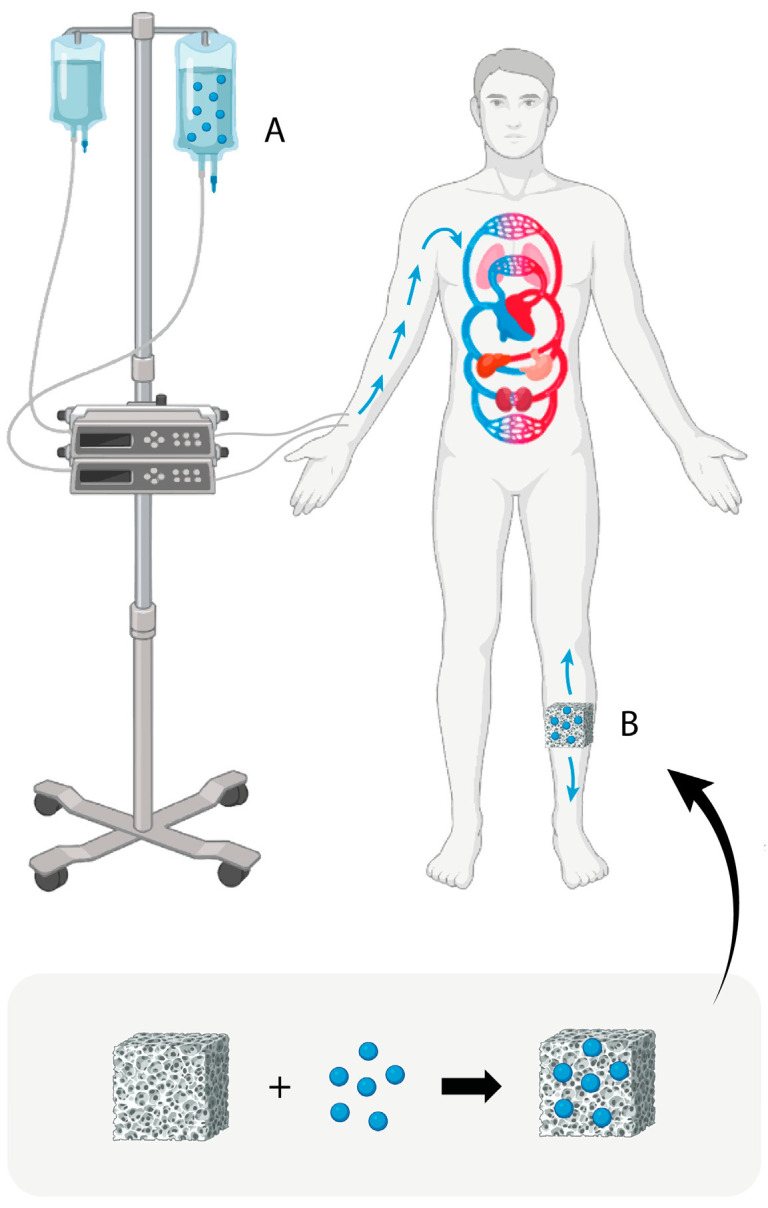
Schematic representation of current antibiotic treatment strategies used to control bone infections (blue spheres: antibiotics; (**A**): intravenous route; (**B**): implantation of a local drug delivery system (DDS) fabricated by loading a scaffold with antibiotics).

The traditional treatment for chronic osteomyelitis includes extensive resection of the infected tissue, repair of the bone defect, and intravenous and local administration of antibiotics over long periods of time [5,6] (Figure 1A). In more specific cases such as infections associated with the implantation of prosthetic materials (hip, knee, and shoulder prosthesis) or osteosynthesis materials for fracture stabilization (plates, screws, pins, etc.), as a first step, treatment requires the simultaneous removal of the implant and intravenous and local antibiotherapy maintenance until normalization of biochemical parameters, as well as the performance of osteoarticular reconstruction surgery as required by the case [5].

Another strategy for the local treatment of chronic bone infections has been based on the administration of antibiotics by means of implantation of drug delivery systems (DDSs) at the site of infection (Figure 1B). One of the most used DDSs for the treatment of osteomyelitis have been antibiotic-loaded poly(methyl methacrylate) (PMMA) beads [7,8]. PMMA, also referred to as (acrylic) bone cement, is a non-resorbable biomaterial that works by slowly releasing antibiotics (generally gentamicin and vancomycin) over time, which can help to eradicate the bacteria causing the infection [9,10]. Despite having been used for decades in clinical practice, PMMA beads or blocks are far from being an ideal antibiotic carrier. The non-degradability of this biomaterial requires a second surgery to remove the beads 2 or 3 weeks after implantation [11].

Vancomycin (Van) is the most commonly used antibiotic in treatment of infections in arthroplasty surgery and chronic osteomyelitis of any etiology [10]. This glycopeptide antibiotic acts primarily as a cell wall synthesis inhibitor in susceptible organisms. It binds rapidly and irreversibly to the cell wall of susceptible bacteria, inhibiting the synthesis of peptidoglycan, which forms the structure of the cell wall [12,13].

Polycaprolactone (PCL) is a synthetic polymer that has been commonly used in 3D-printing applications as a scaffolding component for bone and cartilage reconstruction [14,15,16]. Among its main advantages, we find its biocompatibility, biodegradability, good mechanical properties, and its low melting temperature (≃60 °C), which makes it more versatile than other synthetic polymers used for 3D printing applications [17]. However, the 3D printing temperatures used to fabricate well-defined scaffolds with controlled architecture are around 120–160 °C, which makes it impossible to combine them with cells, growth factors, or other bioactive molecules during the printing process [18,19]. On the other hand, PCL lacks natural motifs that provide specific binding sites for cells that facilitate tissue integration [20]. Because of this, different strategies have been developed to overcome PCL native hydrophobicity, such as surface modification using NaOH treatment or its combination with other synthetic or naturally derived materials (hydrogels) to create hybrid scaffolds with enhanced properties [21,22,23,24]. Synthetic and naturally derived hydrogels have been widely used for different biomedical applications due to their ability to encapsulate cells, drugs, growth factors, or other bioactive molecules [25,26]. Naturally derived hydrogels principally include collagen, chitosan, alginate, silk fibroin, gelatine, cellulose, hyaluronic acid, and hydrogels derived from decellularized tissues [27].

Chitosan (CS) is a naturally derived semicrystalline polymer that is obtained by partial deacetylation of chitin under alkaline conditions [28]. It is one of the materials most widely used to prepare hydrogels due to its excellent biocompatibility, nontoxicity, and biodegradability [29,30]. However, CS hydrogels present several problems due to their unstable structures with large-sized and poorly defined pores, weak formability, and low mechanical properties, limiting their further utilization for in vivo studies [31,32]. For this reason, they have been combined with curable polymers such as polycaprolactone (PCL) and polylactic acid (PLA), which provide the scaffolds basic mechanical support [33,34].

The fabrication of hybrid materials that combine natural and synthetic polymers is a promising approach for creating new scaffolds that combine the intrinsic advantages of both materials and meet several requirements, such as being biodegradable, bioactive, having good mechanical properties, and being easy to fabricate [35]. In this work, we focus on the fabrication of a novel hybrid 3D-printed scaffold based on polycaprolactone and a chitosan hydrogel loaded with different vancomycin concentrations (1%, 5%, 10%, and 20%) as a DDS to evaluate its antimicrobial efficacy against *S. aureus* and *S. epidermidis*. In addition, we propose a novel method for improving the adhesion of hydrophobic polymers (PCL) to hydrogels using two different cold plasma treatments. The obtained scaffolds combine the natural biocompatibility, biodegradability, and antibacterial properties of CS with the excellent mechanical properties of PCL. The morphological characteristics of the scaffolds were studied by means of optical and scanning electron microscopy (SEM), showing that the CS/Van hydrogel successfully coated the PCL matrix homogeneously after the plasma treatments. The antibacterial efficacy of the scaffolds was tested against *Staphylococcus aureus* and *Staphylococcus epidermidis*, and vancomycin release was studied at different time periods by means of high-performance liquid chromatography (HPCL). Finally, we evaluated the possible systemic adverse effects of scaffolds at the cellular level by analyzing the viability, proliferation, and differentiation of a population of adult human bone marrow-derived mesenchymal stem cells (*ah*-BM-MSC).

## 2. Materials and Methods

### 2.1. Materials

Polycaprolactone (PCL) 3D printing filament (molecular weight: 50,000 g/mol) was purchased from 3D4makers (Haarlem, The Netherlands). Chitosan (#448869, 75–85% deacetylated, low molecular weight), acetic acid, and NaOH were obtained from Sigma-Aldrich (Saint Louis, MO, USA) and used as received. Vancomycin hydrochloride was purchased from Lab. Reig Jofre S.A. (Barcelona, Spain).

*Staphylococcus aureus* (CECT 239) and *Staphylococcus epidermidis* (CECT 231) strains were purchased from the Spanish Type Culture Collection (CECT) (Valencia, Spain). Tryptic Soy Broth (TSB), Tryptic Soy Agar (TSA), and buffered peptone water were obtained from Scharlau (Barcelona, Spain).

### 2.2. Methods

#### 2.2.1. Fabrication of Porous Polycaprolactone/Chitosan Scaffolds Loaded with Vancomycin

##### Design and Fabrication of 3D-Printed PCL Scaffolds with Controlled Porosity Using the Fused Deposition Modelling (FDM) Method

**Figure 2:**
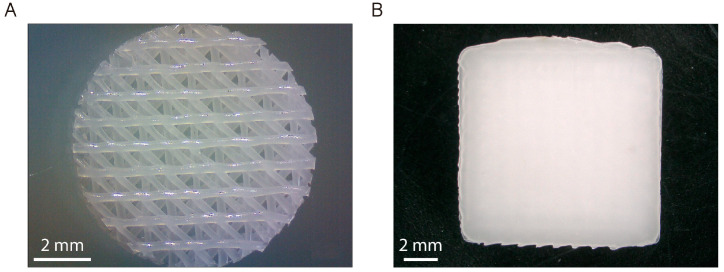
Micrographs of (**A**) porous and (**B**) solid PCL scaffolds printed using REGEMAT 3D Bio V1^®^ bioprinter.

The scaffolds were designed by using REGEMAT 3D Designer software (v1.4.4) and 3D-printed using REGEMAT 3D Bio V1^®^ bioprinter (REG4Life, REGEMAT 3D, Granada, Spain) equipped with a glass bed and a thermoplastic extruder with a 0.4 mm diameter nozzle. The scaffolds were designed with the following parameters: scaffold size 1.50 × 20 × 20 mm (height, width, length), pore size 200 µm, layer height 0.25 mm, perimeters 0, solid bottom/top layers 0, infill pattern triangular; and manufactured using a medical-grade PCL filament printed at 160 °C with an infill speed of 11 mm/s. As described in a previous work, an 8 mm biopsy punch was used to prepare defined and reproducible disk-shaped scaffolds of 8 × 1.50 mm (diameter, height) from each printed scaffold (Figure 2A) [18]. In addition, nonporous (solid) PCL scaffolds of 12 × 12 × 1.50 mm (width, length, height) were printed in order to avoid porosity interfering with some of the experimental results (Figure 2B). Porous disk-shaped scaffolds were characterized and used for all biological and microbiological experiments and solid scaffolds were only used to evaluate the effect of cold plasma treatment on the adhesion of CS/Van hydrogel to the PCL matrix (see Section 2.3.2), as it was noticed that the porosity interfered in the experimental results.

##### Vancomycin-Loaded Chitosan Hydrogel Preparation

The chitosan hydrogels were prepared by dissolving low molecular weight chitosan at a concentration of 4% (*w*/*v*) in demineralized water containing 1.5% (*v*/*v*) acetic acid. The solutions were mechanically stirred for 24 h (250 rpm) and vancomycin was added to the chitosan hydrogels in a content of 1%, 5%, 10%, and 20% (*w*/*w* of chitosan). After 24 h, the solutions were centrifuged (3000 rpm, 1 h) to remove air bubbles.

##### Hybrid Scaffold Preparation

The 3D-printed PCL scaffolds were dip-coated in the chitosan hydrogel and left to dry overnight. Then, the scaffolds were neutralized in a 1 M NaOH solution for 2 h and rinsed three times with distilled water to remove residual acids. The whole process was performed in a laminar flow cabinet to avoid sample contamination.

**Figure 3:**
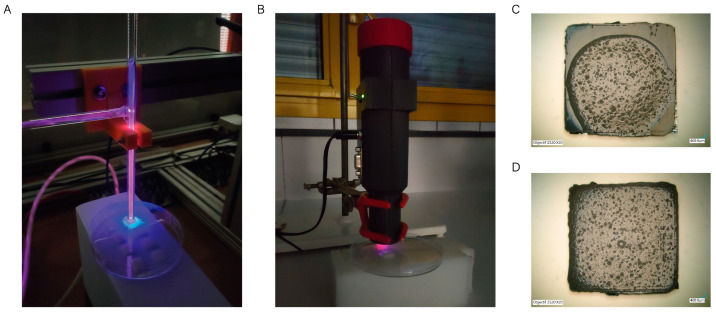
Plasma treatments performed on the PCL scaffolds. (**A**) Cold atmospheric plasma jet; (**B**) homemade torch with a piezoelectric plasma generator. Optical microscopy images of (**C**) Solid PCL scaffold (untreated) coated using CS hydrogel (scale bar 400 µm); (**D**) solid PCL scaffold coated using CS hydrogel after being exposed to cold plasma for 30 s (scale bar 400 µm).

Preliminary tests showed that the chitosan coating remained firmly adhered to the porous PCL scaffolds, as it was trapped between the 3D-printed strands. However, that was not the case for solid PCL scaffolds, which repelled the chitosan coating as seen in Figure 3C. This could be due to the fact that PCL is a hydrophobic polymer, which makes it difficult to adhere to hydrogels with more than 90% water content.

Plasma treatments have recently been used to lower the hydrophobicity of polymer surfaces by forming reactive oxygen species (ROS) [36]. These newly formed reactive species generate hydrophilic groups (hydroxyl or carboxylic) on the polymer surface that can interact with the hydrogels [37]. Even if plasma treatment was not necessary for porous scaffolds, two different plasma technologies were tested on the surfaces of solid PCL scaffolds: (i) a cold atmospheric pressure plasma jet developed in GREMI-CNRS consisting of a plasma gun that is a dielectric barrier discharge (DBD) plasma and helium fed and powered using a microsecond voltage pulse [38] (Figure 3A); and (ii) a homemade torch with a piezoelectric plasma generator (CeraPlas^®^) developed by TDK Electronics GmbH & Co [39] (Figure 3B). This technology is based on a piezo ceramic component and a driving circuit that allows for generating cold atmospheric plasma. After being exposed for 30 s to plasma treatments with 5 mm gap, the PCL scaffolds were dip-coated in the chitosan hydrogels and left to dry overnight. As can be seen in Figure 3D, the CS hydrogel was not repelled by the scaffold, showing a homogeneous distribution.

In order to determine the amount of chitosan adhered to the scaffolds, the weight of the PCL scaffolds was compared to the weight of PCL/CS/Van scaffolds after the coating process. After weighing the scaffolds (n = 10) in an analytical balance, an increment from 33.34 ± 0.34 mg (native PCL) to 41.16 ± 1.01 mg (PCL/CS/Van scaffolds) was observed. No significant differences were obtained between the weight of PCL/CS scaffolds at different vancomycin concentrations.

### 2.3. Characterization of the 3D-Printed Scaffolds

#### 2.3.1. Morphological Characterization of the 3D-Printed Scaffolds

The morphological characterization of the 3D-printed scaffolds was carried out using a digital camera coupled to a stereomicroscope. The surface morphology of the scaffolds was examined using scanning electron microscopy (SEM; model JEOL-6100, JEOL Ltd., Tokyo, Japan). Prior to visualization, the scaffolds were mounted on aluminum stubs with conductive paint and sputter-coated with platinum (10 mA, 120 s). SEM micrographs were obtained at magnifications of ×20 and ×45 under an accelerating voltage of 15–20 kV and a working distance of 4–5 mm.

#### 2.3.2. Wettability Assay

Static water contact angle (WCA) measurements were performed on a drop shape analyzer (DSA100S KRÜSS GmbH) to assess the effect of cold plasma treatment on PCL hydrophobicity. The water droplet volume was set to 1 µL and the deposit speed at 2.67 µL/s. Briefly, solid PCL scaffolds (n = 3) were treated using a cold atmospheric plasma jet (Plasma 1) and a homemade torch with a piezoelectric plasma generator (Plasma 2) (Figure 3). Three untreated scaffolds were used as control. Each measurement was performed in triplicate in three different scaffolds for better statistical significance.

#### 2.3.3. In Vitro Degradation Kinetics

The in vitro degradation of the scaffolds was assessed by measuring the weight loss of the constructs at 1, 3, 7, 14, 21, and 28 days. Briefly, PCL/CS/Van scaffolds were weighed (W_0_, initial weight) and subsequently immersed in eppendorf tubes containing 1 mL of complete growth medium (DMEM). Then, the scaffolds were incubated at 37 °C in a 5% CO_2_ atmosphere at 95% relative humidity. At different time periods, the scaffolds were recovered from medium, sightly wiped using filter paper, and reweighed (W_d_, final weight). The weight loss (WL) was calculated by applying the following equation [40]:

WL% = [(W_0_ − W_d_)/W_0_] × 100 (1)

where W_0_ and W_d_ indicate the weight of the scaffold before and after the scheduled immersion time, respectively.

#### 2.3.4. Water Absorption Assay

The swelling behavior of PCL/CS/Van scaffolds was tested in PBS solutions of pH 7.4. Three PCL/CS/Van scaffolds of known weight were put into stainless steel baskets in order to prevent scaffolds from floating. At different timepoints (0.5, 1, 3, 6, 24, 48 h), the scaffolds were collected and gently wiped using filter paper before being weighed. Water absorption percentage was calculated using the following equation [41]:

(2) $$Water\;absorption\;\left(\%\right)=\frac{w_{t}-w_{0}}{w_{0}}\times 100$$

where $w_{t}$ is the weight of the scaffolds at time *t*, and $w_{0}$ is the initial weight of the dry scaffolds.

#### 2.3.5. Vancomycin Release Quantification

Vancomycin-loaded chitosan scaffolds were immersed into 1.5 mL eppendorf tubes containing 1 mL phosphate-buffered saline (PBS, pH 7.4) medium at 37 °C in darkness. At predetermined timepoints (1 h, 3 h, 6 h, 24 h, 48 h, 72 h, 7 d, and 14 d), the medium was collected, and fresh PBS was refilled accordingly. The vancomycin concentration was measured using high-performance liquid chromatography (HPLC) (Agilent 1200 series, Agilent Technologies, Waldbronn, Germany) with diode array (DAD) and fluorescence (FLD) detector, equipped with a Vydac 218TP C18 column (250 × 4.6 mm, 5 μm, Avantor^®^, Radnor Township, PA, USA). The analysis was performed under isocratic conditions (0.8 mL/min) using 89% ammonium phosphate 0.5 M and 11% acetonitrile as mobile phase. Prior to the measurements, a calibration curve of vancomycin in PBS was determined by measuring absorbance values of stock solutions of vancomycin from 100 to 0.5 μg/mL at 282 nm (linear range 12.4 to 0.5 μg/mL; R^2^ > 0.998).

### 2.4. Evaluation of Antimicrobial Properties

#### 2.4.1. Bacterial Culture

Strains of *Staphylococcus aureus* (CECT 239) and *Staphylococcus epidermidis* (CECT 231) were obtained from the Spanish Type Culture Collection (CECT) (Valencia, Spain). To activate the *S. aureus* and *S. epidermidis* strains, they were grown in TSB medium and incubated aerobically at 35 °C for 24 h. The bacterial cultures were stored in TSA medium at 4 °C for over three months. For daily use, a working culture was prepared by transferring a single colony from TSA to 10 mL of TSB, followed by incubation at 35 °C for 24 h.

#### 2.4.2. Agar Diffusion Method

In order to assess the antimicrobial properties of the chitosan coating loaded with vancomycin, experiments were conducted to measure the inhibition zones. Initially, a suspension of each microorganism (*S. aureus* and *S. epidermidis*) was prepared with a concentration of 5.0 log10 colony-forming units/mL (CFU/mL) in TSB (2X) medium. Subsequently, 100 µL aliquots of the bacterial suspension were added to 6.0 mm diameter well plates containing TSA agar.

Following a brief drying period, the samples were brought into contact with the PCL/Chitosan/Van scaffolds and incubated overnight at 37 °C. Finally, the inhibition halos were measured using a ruler at 24 and 48 h. All the experiments were replicated three times.

### 2.5. Biological Assays

#### 2.5.1. Isolation, Purification, Characterization, and Culture of Adult Human Bone Marrow-Derived Mesenchymal Stem Cells (*ah*-BM-MSCs)

Multipotent *ah*-BM-MSCs were isolated from bone marrow as previously described (additional information related to the applied methodology, cell isolation, culture, and expansion can be found in previous publications [42,43]). Briefly, bone marrow was collected from the iliac crest of three volunteer patients by direct puncture. Then, bone marrow aspirate was transferred into transfer bags containing heparin. The mononuclear cell fraction was obtained using Ficoll density gradient media and a cell-washing closed automated SEPAX^TM^ S-100 system (Biosafe, Eysines, Switzerland). After assessing cell viability by using trypan blue staining (#T8154; Sigma-Aldrich, Saint Louis, MO, USA), cells were plated out at a density of 3.75 × 10^5^ mL in 75 cm^2^ culture flasks (Sarstedt, Nümbrecht, Germany) with 10 mL of basal culture growth medium (GM). The growth medium used was Dulbecco’s Minimum Essential Media (DMEM) (#31885-023, Gibco, Bleiswijk, The Netherlands) complemented with 10% (*v*/*v*) inactivated fetal bovine serum (FBS) (#F7524, Sigma-Aldrich, Saint Louis, MO, USA) and routine antibiotics such as Penicillin and streptomycin (100 U mL^−1^ penicillin and 100 μg mL^−1^ streptomycin) (#P4333, Sigma-Aldrich, Saint Louis, MO, USA) before incubating cells under standard conditions of normoxia (21% O_2_) at 37 °C and 5% CO_2_. *ah*-BM-MSCs in passages 3–5 were used for in vitro experiments.

All biological experiments were in full compliance with the established regulations and the experimental protocol was reviewed and approved by the Institutional Ethics Committee of UCAM—Universidad Católica de Murcia (authorization nº CE051904) UCAM Ethics Committee (CE nº 052114/05.28.2022).

#### 2.5.2. Immunophenotypic Profiles of *ah*-BM-MSC Cultures

The isolated *ah*-BM-MSCs were characterized by means of flow cytometry (Beckman Dickinson & Co., Franklin Lakes, NJ, USA; Navios Software v1.2) for mesenchymal (CD90, CD73, 105) and hematopoietic (CD34, CD45) markers as previously described [42,44,45]. Before performing the in vitro assays, single-cell suspensions obtained using culture trypsinization were labeled with fluorochrome-conjugated antibodies: CD73-PE, CD90-APC, CD105-FITC, CD34-APC, and CD45-FITC (Human MSC Phenotyping Cocktail, Miltenyi Biotec, Bergisch Gladbach, Germany) in order to verify the purity of *ah*-BM-MSCs populations.

#### 2.5.3. Cell Seeding Methods

For cell viability, proliferation, and osteogenic differentiation assays, *ah*-BM-MSCs were seeded at the bottom of 24 WP at a density of 10 × 10^3^ cells cm^−2^, and PCL/CS/Van scaffolds were placed in 0.4 μm pore culture well inserts (Falcon^®^) to be in indirect contact with the culture. Cells seeded onto tissue culture-treated polystyrene wells (TCPS; Sigma-Aldrich, Corning, NY, USA) (without materials) were taken as positive control. The culture media were changed three times a week for all the experiments performed.

#### 2.5.4. Cell Viability and Proliferation Assays

##### In Vitro Cytotoxicity Assay

**Figure 4:**
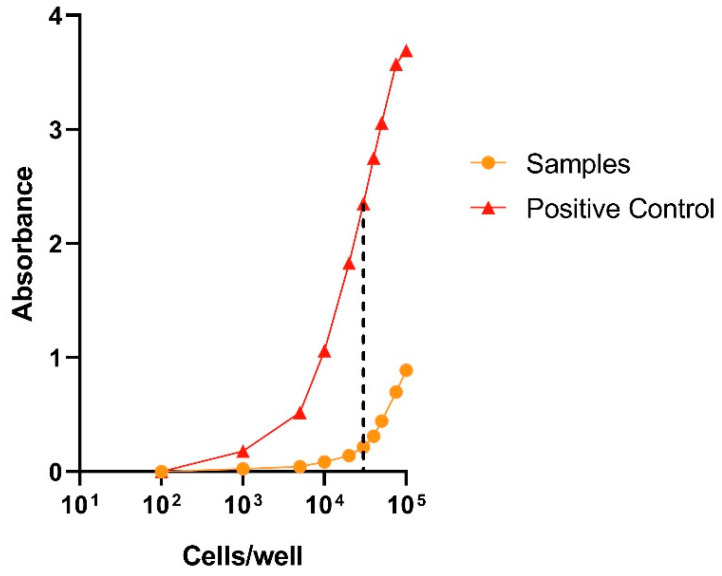
Determination of the optimal target cell concentration for *ah*-BM-MSCs. *ah*-BM-MSCs were titrated in 24 WP at densities of 100, 1000, 5000, 10,000, 20,000, 30,000, 40,000, 50,000, 75,000, and 100,000 cells/well. Culture medium was added for the determination of the spontaneous LDH activity (samples), and culture medium containing 1% Triton X-100 was added to determine the maximum LDH release (positive control).

The cell viability of *ah*-BM-MSCs was evaluated using LDH cytotoxicity detection kit (#11644793001, Roche Diagnostics, Roche. Applied Science, Mannheim, Germany) after placing PCL/CS/Van scaffolds in indirect contact with cells for 24 and 72 h. Before performing the assay, it is important to determine the optimal cell concentration for the cell line used, as different cell types may contain different amounts of LDH. In general, we choose the concentration at which the difference between the low and high control is at a maximum. For our culture of *ah*-BM-MSCs, the optimal cell concentration to be used for the subsequent experiment was found to be 3 × 10^4^ cells/well for 24 WP (Figure 4).

Twenty-four hours after seeding *ah*-BM-MSCs on 24 WP, the culture medium was replaced using fresh assay medium containing 1% FBS to remove LDH activity released from the cells during the incubation step. Then, the PCL/CS/Van scaffolds were placed in indirect contact with cells using 0.4 μm pore culture well inserts and LDH activity was assessed at 24 and 72 h. Cells growing on tissue culture polystyrene (TCPS) plates without PCL/CS/Van scaffolds were used as low control (spontaneous LDH release) and cells cultured using assay medium containing 1% Triton X-100 solution were used as high control (maximum LDH release). At different timepoints, 100 μL aliquots of each well were transferred to an optically clear flat-bottomed 96 WP followed by the addition of 100 μL of the reaction mixture. After 30 min incubation at RT, the absorbance was read directly using a Spectramax iD3 plate reader (Molecular Devices, San Jose, CA, USA) at 490 nm.

##### Cellular Metabolic Activity Assay

The cellular metabolic activity of *ah*-BM-MSCs grown in the presence of PCL/CS scaffolds loaded with different vancomycin concentrations was evaluated using the AlamarBlue^®^ assay (#DAL1100, Invitrogen, Carlsbad, CA, USA) on days 1, 3, 7, 14 after seeding. Cells growing onto TCPS (without PCL/CS/Van scaffolds) were used as control. Briefly, at different time periods, the inserts containing PCL/CS/Van scaffolds were temporarily removed (prior to the incubation step), and fresh medium (1 mL) containing 10% (*v*/*v*) AlamarBlue^®^ reagent was added to each well. Then, the plate was incubated for 4 h in darkness (wrapped with aluminum foil) at 37 °C in a 5% CO_2_ atmosphere with 95% of relative humidity. Finally, 150 μL aliquots of each well were transferred to a black-walled 96 WP and fluorescence was assessed at excitation and emission wavelengths of 530 and 590 nm (respectively) using a Spectramax iD3 plate reader (Molecular Devices, USA).

#### 2.5.5. Osteoblastic Differentiation Assays

##### Alkaline Phosphatase (ALP) Activity

The ALP activity of *ah*-BM-MSCs was assessed at 7 and 14 days using an Alkaline Phosphatase Detection Kit (#SCR004, Merck Millipore, Billerica, MA, USA) after seeding the cells in the presence of PCL, PCL/CS, and PCL/CS/10%Van scaffolds. At each time period, cells were detached using trypsin, and an aliquot of 1.5 × 10^4^ cells (per sample) was treated by adding 100 μL p-nitrophenylphosphate (p-NPP). After 20 min incubation at RT, the catalytic reaction (p-NNP → phosphate + p-nitrophenol) affords a yellow colored byproduct that is proportional to the amount of ALP present within the reaction. The absorbance was measured using a Spectramax iD3 plate reader (Molecular Devices, USA) at a wavelength of 405 nm.

##### In Vitro Mineralization Assay

In vitro mineralization was evaluated using an Osteogenesis Assay Kit (#ECM815; Millipore, Billerica, MA, USA) 21 days after seeding *ah*-BM-MSCs in the presence (indirect contact) of PCL, PCL/CS, and PCL/CS/10%Van scaffolds. Briefly, the *ah*-BM-MSCs were fixed using 10% formaldehyde, stained using alizarin red stain solution, and visualized using an optical microscope (Axio Vert.A1, ZEISS, Jena, Germany) coupled to a digital camera (Axiocam 305 color, ZEISS, Germany). Then, the samples were treated following the manufacturer’s protocol to quantify matrix mineralization and the optical density (OD) was measured using a Spectramax iD3 plate reader (Molecular Devices, USA) at 405 nm.

### 2.6. Statistics

All data were represented as mean ± SD, n = 3. The statistical significance was determined by a two-way ANOVA using GraphPrism 8.0.1 (GraphPad Software Inc., San Diego, CA, USA) for Windows. Comparison between groups was evaluated using the *t*-test, the significance level being *p* < 0.05.

## 3. Results and Discussion

### 3.1. Characterization of the 3D-Printed Scaffolds

#### 3.1.1. Morphological Characterization of the 3D-Printed Scaffolds

**Figure 5:**
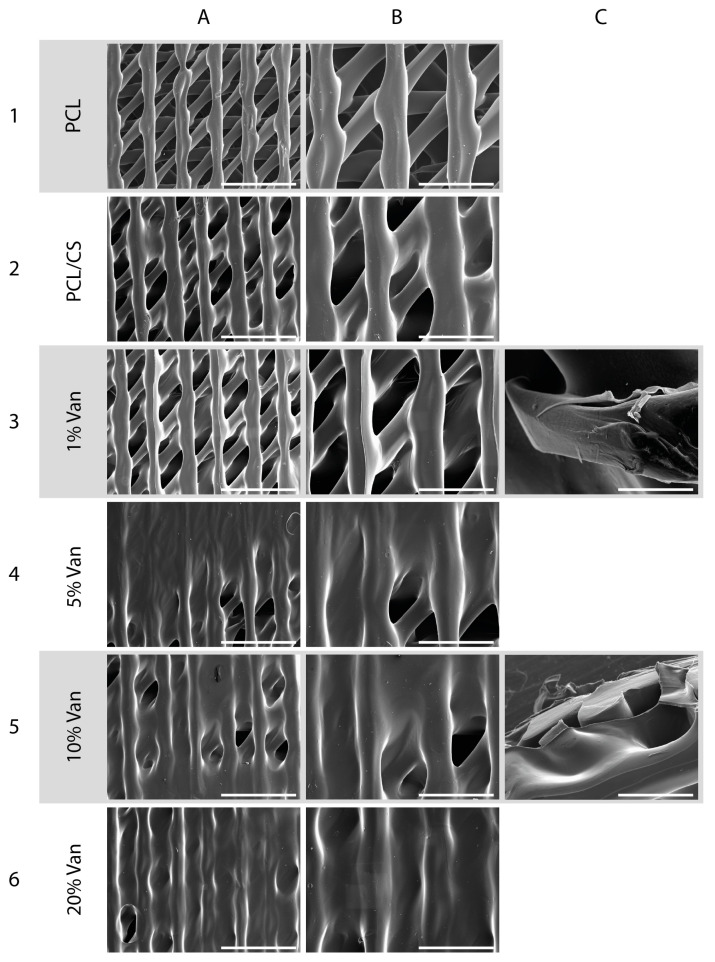
SEM micrographs of the (**1**) PCL, (**2**) PCL/CS, and (**3**–**6**) PCL/CS/Van scaffolds loaded with 1%, 5%, 10%, and 20% *w*/*t* vancomycin, respectively, at different magnifications: (**A1**–**A6**) 22×, scale bar 2 mm; (**B1**–**B6**) 40×, scale bar 1 mm; (**C3**) scale bar 200 µm; (**C5**) scale bar 700 µm.

Microstructural examination of the scaffolds’ morphology using SEM imaging showed differences in the scaffolds’ surfaces depending on the vancomycin concentration loaded. Figure 5(A1,B1) shows a micrograph of the native PCL constructs before being coated with chitosan hydrogel. The scaffolds show a parallel distribution of the printed strands in each printed layer, and the triangular printing pattern results in scaffolds with interconnected porosity with an approximate pore size of 200 µm and an overall porosity of 59%, as calculated in a previous publication [18]. As can be seen in Figure 5(A2,B2), the chitosan coating causes a discrete thickening of the 3D-printed strands as well as at the points of contact between the strands as a result of the thin coating layer provided by CS. However, this finding intensifies progressively and proportionally when the chitosan hydrogel is loaded with increasing vancomycin concentrations (Figure 5(A3–A6,B3–B6)). The latter is observed in the micrographs corresponding to PCL/CS/20%Van scaffolds, in which almost the entire surface of the PCL scaffold was covered, making it difficult to distinguish its pores (Figure 5(A6,B6)).

The functional characteristics of the construct developed in this study agree with the premises established by different authors for the design and fabrication of 3D scaffolds to be used for tissue engineering applications [46,47]. In fact, we have taken into account that an ideal construct or scaffold, in general, should have sufficient porosity to accommodate both differentiated and undifferentiated cells of different lineages. In addition, it was combined with a natural-derived polymer (chitosan) capable of acting as a carrier or DDS for the controlled release of drugs or biomolecules. Likewise, these reticular pore systems should have open and interconnected porosity to facilitate the diffusion of nutrients, metabolites, and cells from the external environment to the inner parts of the scaffold [20].

#### 3.1.2. Wettability Assay

**Figure 6:**
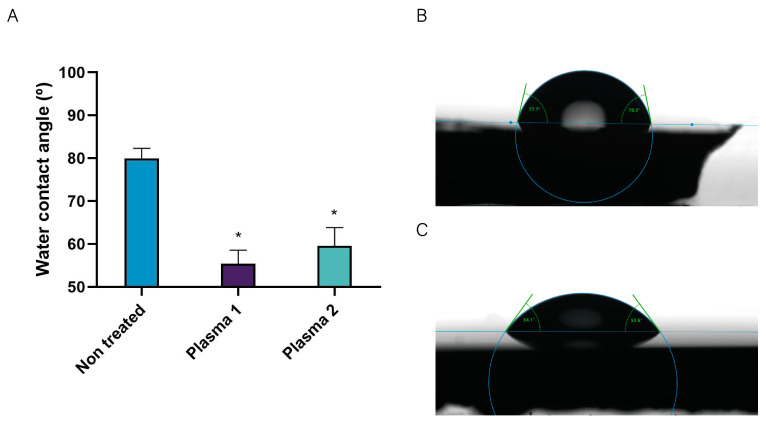
(**A**) Water contact angle on 3D-printed solid (nonporous) PCL scaffolds before and after plasma treatments (Plasma 1: cold atmospheric pressure plasma jet; Plasma 2: homemade torch with piezoelectric plasma generator). Water droplets with a volume of 1 µL deposited on (**B**) nontreated PCL scaffolds and (**C**) PCL scaffolds treated using Plasma 1. Data represent mean ± SD. * Significant differences with the water contact angle of nontreated PCL scaffold.

The wettability of solid 3D-printed PCL scaffolds was evaluated by measuring the static water contact angle of 1 µL water droplets before and after the plasma treatments. As shown in Figure 6, the static WCA of the untreated PCL scaffolds was 79.93 ± 2.36°, demonstrating hydrophobic behavior without any surface modification. However, for plasma-activated scaffolds, we observed water contact angles of 55.40 ± 3.16° and 59.62 ± 4.22° for Plasma 1 and Plasma 2, respectively, showing a decrease of around 20° that attests to a decrease in surface hydrophobicity.

A change in WCA is a clear indicator of a polymer surface chemistry modification [48]. The results obtained are in total accordance with the ones obtained by Carette et al., who studied the effects of atmospheric plasma on the surface of a polylactic acid (PLA) scaffold in order to improve the adhesion of a 1% chitosan solution, obtaining a decrease of about 30° in WCA (from 85° to 55°) after plasma activation [36].

#### 3.1.3. In Vitro Degradation Kinetics

The in vitro degradation assay carried out using PCL/CS/Van scaffolds did not result in any changes in the scaffold weights (W_0_ = W_d_) after 28 days of immersion in culture medium at 37 °C in a 5% CO_2_ atmosphere at 95% relative humidity. It is well known that CS, as well as other polysaccharides, is degraded in the body by depolymerization, oxidation, and hydrolysis (enzymatic or nonenzymatic) [49]. Lim et al. studied the degradation of CS porous beads with different degrees of acetylation (DA 10–50%) in different enzyme solutions (lysozyme, NAGase, and a mixture of both) [50]. As main findings, they determined that (i) CS beads did not degrade in the NAGase solution, (ii) CS beads with high DA degraded faster than those with low DA in lysozyme and enzymatic mixture solutions, and (iii) CS beads with DA values around 30 to 50% rapidly degraded into monomers in the enzymatic mixture in less than 30 to 60 days.

#### 3.1.4. Water Absorption Assay

**Figure 7:**
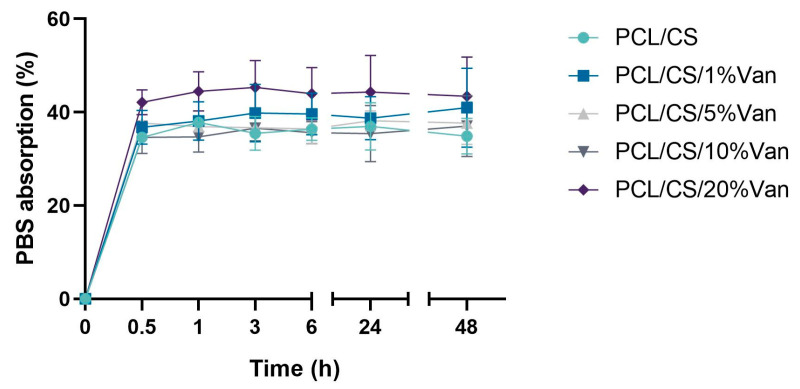
Water absorption as a function of time of PCL/CS and PCL/CS/Van scaffolds (1%, 5%, 10%, and 20%) in contact with PBS medium (pH = 7.4).

The water absorption of the chitosan coating of PCL/CS and PCL/CS/Van scaffolds was tested in PBS solutions. As can be seen in Figure 7, the total weight of the scaffolds increased between 34.61 ± 1.13% (PCL/CS) and 42.14 ± 1.46% (PCL/CS/20%Van) after 30 min of immersion in PBS, and remained more or less stable for at least for 48 h. The minimum absorption values were shown by the PCL/CS scaffolds (34.61 ± 1.13%), while the maximum ones were reached by the PCL/CS/20%Van scaffolds 45.32 ± 8.41%. The latter can be justified by the results obtained in the scaffolds characterization section (SEM imaging), which showed that increasing concentrations of vancomycin caused a thickening of the 3D-printed strands, having more chitosan surface to absorb PBS.

#### 3.1.5. Vancomycin Release Quantification

**Figure 8:**
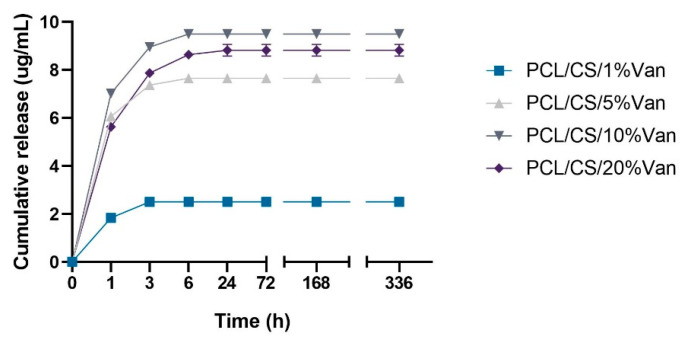
In vitro cumulative drug release profiles of PCL/CS scaffolds loaded with 1%, 5%, 10%, and 20% vancomycin for 14 days (336 h).

The vancomycin release from the PCL/CS scaffolds loaded with 1%, 5%, 10%, and 20% vancomycin followed first-order release kinetics (Figure 8). Fast vancomycin release was observed during the first hour, followed by slower release during the next hours for all the scaffolds studied. As can be seen in the graph, vancomycin release finishes after 24 h and the cumulative drug release profiles seem to reach a plateau. These release profiles could be explained by the presence of two different drug fractions: (i) drug weakly bound or deposited on the CS hydrogel surface, and (ii) drug included in the CS network by chemical interactions. It is worth noting that the scaffolds loaded with 10% and 20% Van had similar drug release profiles, indicating that the solubility limit of the drug in PBS is probably between both concentrations.

These kinetics profiles are in total accordance with the results obtained by López-Iglesias et al., who studied vancomycin release from porous chitosan aerogels obtaining similar drug release profiles [51]. On the other hand, Kausar et al. developed chitosan films loaded with vancomycin (10 and 20% with respect to dry polymer mass), obtaining 100% dissolution of free vancomycin in 7 h with more than 50% of drug dissolved within the first hour [52].

### 3.2. Evaluation of Antimicrobial Properties

**Figure 9:**
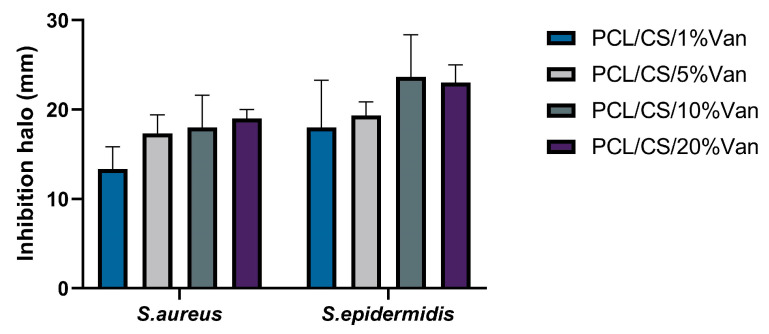
Inhibition halos (mm) produced by PCL/CS/Van scaffolds loaded with 1%, 5%, 10%, and 20% *w*/*t* vancomycin for *S. aureus* and *S. epidermidis*. Data represent mean ± SD. No significant differences were found between the scaffolds for both microorganisms studied.

PCL/Chitosan scaffolds loaded with different vancomycin concentrations have been shown to exhibit antimicrobial activity against both *S. epidermidis* and *S. aureus*. This is because, in all cases, the minimum inhibitory concentration for vancomycin was exceeded (2 μg/mL for *S. aureus* and 2–4 μg/mL for *S. epidermidis*) [53,54]. The inhibition halos were measured at 24 and 48 h, with no significant differences being observed between them. This may be due to the fact that no more vancomycin is released between 6 and 24 h (Figure 9).

As seen in Figure 9, a halo of inhibition is shown in all cases. In the case of *S. aureus*, the highest inhibition halo is for PCL/CS/20%Van, but there are no significant differences with the PCL/CS/10%Van and PCL/CS/5%Van scaffolds. This is because the vancomycin concentrations in the media are similar to each other, 9.5 mg/mL and 8.82 mg/mL (Figure 9), respectively. In the case of *S. epidermidis*, the highest inhibition halo was obtained for the PCL/CS/10%Van scaffolds. If the PCL/CS/10%Van and PCL/CS/20%Van scaffolds are compared, there are no significant differences between them because the concentrations of vancomycin in the media are similar (Figure 9).

If we compare the inhibition halos of *S. epidermidis* and *S. aureus*, it is observed that although both bacteria are Gram-positive; vancomycin is more effective against *S. epidermidis*. No antimicrobial effect of PCL/CS was observed, this may be due to the low solubility and the limited amount of positive charges of the polysaccharide at the pH (7.4 ± 0.1) of the culture medium [55,56].

### 3.3. Biological Assays

#### 3.3.1. Cell Viability and Proliferation Assays

##### In Vitro Cytotoxicity Assay

**Figure 10:**
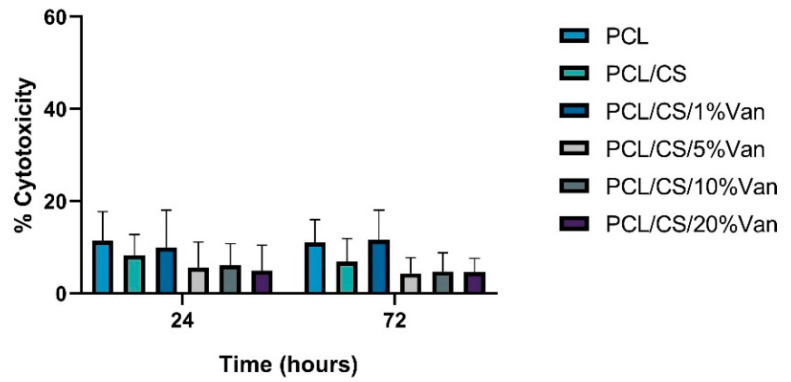
Cytotoxicity results using the LDH assay after placing PCL, PCL/CS, and PCL/CS/Van scaffolds in indirect contact with *ah*-BM-MSCs for 24 and 72 h. The mean cytotoxicity percentage was calculated and normalized with respect to spontaneous LDH release (low control) and maximum LDH release (high control). Bars represent standard deviations of the mean. No significant differences were observed between the tested samples.

Cytotoxicity was assessed using an LDH activity assay after placing the PCL/CS/Van scaffolds in indirect contact with *ah*-BM-MSCs for 24 and 72 h (Figure 10). The results of LDH release demonstrated that none of the tested scaffolds were found to be cytotoxic. However, the maximum LDH release values were achieved by PCL, PCL/CS, and PCL/CS/1%Van scaffolds, showing cytotoxicity values of 11.4 ± 6.4%, 8.3 ± 4.5%, and 10.0 ± 8.0% at 24 h, and 11.1 ± 4.9%, 6.9 ± 5.0%, and 11.7 ± 6.4% at 72 h, respectively, with respect to low and high control. On another hand, PCL/CS scaffolds loaded with 5, 10, and 20% vancomycin reached cytotoxicity values of 5.7 ± 5.5%, 6.1 ± 4.7%, and 4.9 ± 5.5% at 24 h, and 4.4 ± 3.4%, 4.8 ± 4.0%, and 4.7 ± 2.9% at 72 h, respectively. No statistically significant differences were observed between the tested samples.

Both polymers used in this study (PCL and CS) have been commonly used as DDSs or scaffolds components for tissue engineering applications due to their biocompatibility, biodegradability, and ease of modification [57,58]. Cytocompatibility of CS has already been studied, with results showing that it does not have an acute cytotoxic effect [59]. Our results are in line with previous publications that demonstrate the good biocompatibility of PCL and CS-based structures [33,60].

##### Cellular Metabolic Activity Assay

**Figure 11:**
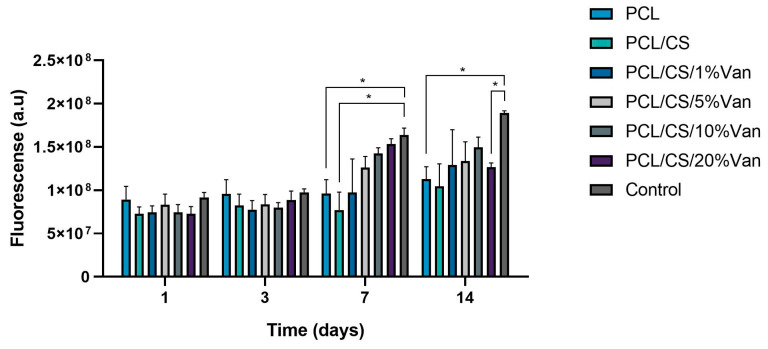
Cellular metabolic activity of *ah*-BM-MSCs using the AlamarBlue^®^ assay at different time periods. The metabolic activity of cells seeded on plastic (TCPs) was used as positive control. Bars represent standard deviations of the mean. * Significant differences between the bracketed groups at the same time period.

The cellular metabolic activity of *ah*-BM-MSCs growing in the presence of PCL, PCL/CS, and PCL/CS/Van scaffolds was evaluated using the AlamarBlue^®^ assay on days 1, 3, 7, and 14 after seeding (Figure 11). As can be seen in the graph, the metabolic activity of *ah*-BM-MSCs increased gradually from days 1 to 14 for all the scaffolds studied except for PCL/CS/20%Van, in which a slight decrease was observed after day 7. No significant differences were observed at early periods (days 1 and 3), where all the scaffolds showed a similar proliferation rate. On the other hand, significant differences were noted between PCL, PCL/CS scaffolds, and the control on day 7, and between PCL, PCL/CS/20%Van, and the control on day 14. It is worth noting that the PCL/CS/10%Van scaffolds showed higher metabolic activity with respect to the scaffolds studied.

#### 3.3.2. Osteoblastic Differentiation Assays

Osteogenic differentiation was assessed on PCL, PCL/CS, and PCL/CS/10%Van scaffolds, as it provided the best results in the cytotoxicity and proliferation assays, as well as in the microbiological assays, showing a similar bactericidal effect to the scaffold loaded with 20% vancomycin.

##### Alkaline Phosphatase (ALP) Activity

**Figure 12:**
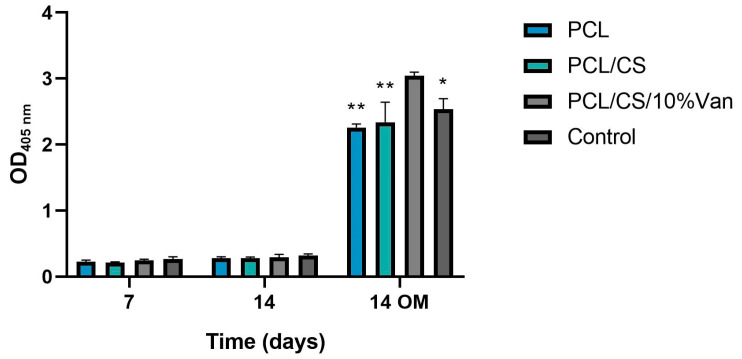
Alkaline phosphatase activity of *ah*-BM-MSCs after being cultured for 7 and 14 days in the presence of PCL, PCL/CS, and PCL/CS/10%Van scaffolds using growth medium (GM) and osteogenic medium (OM). Cells seeded on plastic (TCPs) were taken as positive control. Results are shown as a function of optical density (OD_405nm_) units. Data represent mean ± SD. Significant differences were found between PCL/CS/10%Van and the other samples at 14 days using OM; * *p* < 0.001 and ** *p* < 0.0001.

The Alkaline Phosphatase activity of *ah*-BM-MSCs cultured in the presence of PCL, PCL/CS, and PCL/CS/10%Van scaffolds is shown in Figure 12. From days 7 to 14, using growth medium (GM), no significant differences in ALP activity were observed between the different time periods or within the scaffolds studied. On the other hand, when using osteogenic medium (OM), the ALP activity significantly increased at 14 days, reaching the maximum values in the presence of PCL/CS/10%Van. In fact, significant differences were observed between the scaffold loaded with 10% vancomycin and the other experimental groups, indicating that PCL/CS/10%Van seems to increase the ALP activity of the cultured cells.

However, the PCL/CS scaffold did not show an increase in ALP activity, so this finding cannot be attributed to the presence of the chitosan coating. Other authors have followed novel strategies to increase the osteogenic activity of chitosan, consisting of its combination with hydroxyapatite (HA) via the fabrication of carboxymethyl chitosan–HA nanofibers [61] or HA/resveratrol/chitosan composite microspheres [62]. This kind of HA composite can provide necessary calcium sources that can consequently induce cell differentiation, deposition of extracellular matrix, and mineralization [63].

##### In Vitro Mineralization Assay

**Figure 13:**
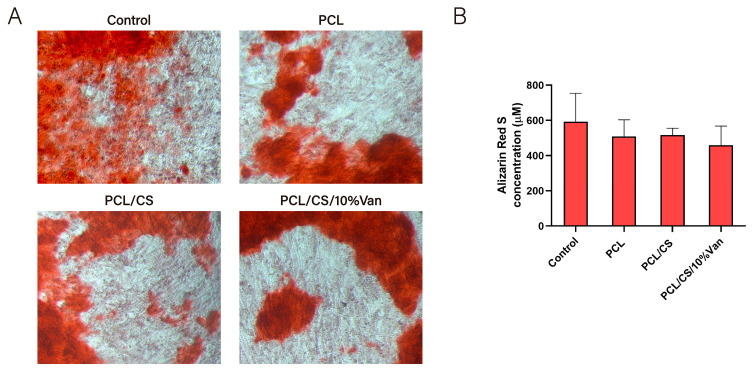
Alizarin red staining of *ah*-BM-MSCs after being cultured for 21 days in the presence of PCL, PCL/CS, and PCL/CS/10%Van scaffolds. (**A**) Alizarin red staining showing mineralization (original magnification ×10); (**B**) quantitative determination of alizarin red staining. Cells seeded on plastic (TCPs) were taken as positive control. Quantitative data are presented as mean ± SD. No significant differences were observed between groups.

The osteogenic differentiation of *ah*-BM-MSCs was determined by quantifying the presence of calcium deposits after placing the PCL/CS/Van scaffolds in indirect contact with *ah*-BM-MSCs for 21 days. As shown in Figure 13A, no major visual differences were observed between the samples, although the control appears to have a slightly higher reddish coloration. Quantitative examination (Figure 13B) showed that the alizarin red staining concentration was higher in the control than in the cells exposed to the scaffolds, even if no significant differences were observed between the samples. This could be due to a cellular adaptation phase to varying environmental changes caused by the presence of the scaffolds, which could have slightly delayed or affected mineralization.

As demonstrated by both osteoblastic differentiation assays, the PCL, PCL/CS, and PCL/CS/10%Van scaffolds did not affect *ah*-BM-MSCs differentiation capacity, indicating that both the scaffold components and the vancomycin dose used did not cause adverse effects or alterations at the cellular level.

## 4. Conclusions

Biocompatible 3D-printed hybrid PCL/CS/Van scaffolds were developed as drug delivery systems with antimicrobial efficacy against *S. aureus* and *S. epidermidis*. Two cold plasma treatments were presented as novel methods to improve the adhesion of hydrophobic polymers to hydrogels, resulting in a decrease in PCL water contact angle of 20°. All the scaffolds studied (1%, 5%, 10%, and 20%Van) had antimicrobial activity against both *S. aureus* and *S. epidermidis*, showing higher inhibition halos for PCL/CS/10%Van and PCL/CS/20%Van scaffolds. In vitro assays demonstrated that the PCL/CS/Van scaffolds were found to be biocompatible and bioactive as demonstrated by no cytotoxicity or functional alteration of the cultured *ah*-BM-MSCs. In conclusion, the developed DDS could be useful in achieving a controlled and effective local release of vancomycin against *S. aureus* and *S. epidermidis*, considered as the main causes of bone infections, but further preclinical studies in vivo using animal models are needed to confirm these results.

## Data Availability

The data presented in this study are disclosed in the main text.
